# Small Activating RNAs: A Curated Database and an Overview of Rational Design Principles

**DOI:** 10.3390/molecules31183249

**Published:** 2026-09-14

**Authors:** Petra Sušjan-Leite, Peter Pečan, Ana Godeša, Fabienne Ramona Caldana, Roman Jerala

**Affiliations:** 1 Department of Synthetic Biology and Immunology, National Institute of Chemistry, Hajdrihova ulica 19, 1000 Ljubljana, Slovenia; peter.pecan@ki.si (P.P.); ana.godesa@ki.si (A.G.);; 2 Graduate School of Biomedicine, University of Ljubljana, Vrazov trg 2, 1000 Ljubljana, Slovenia; 3 Centre for Technology of Gene and Cell Therapy, National Institute of Chemistry, Hajdrihova ulica 19, 1000 Ljubljana, Slovenia

**Keywords:** RNA activation, small activating RNAs, annotated database, rational design

## Abstract

RNA activation (RNAa), mediated by small activating RNAs (saRNAs), upregulates endogenous gene expression through recruitment of the Argonaute proteins to the promoter, natural antisense transcripts, or other noncoding RNAs. This mechanistically distinct modality addresses an unmet need, as few RNA therapeutics currently function to activate endogenous gene expression. Three saRNA therapeutics are now in clinical trials; however, broader application remains constrained by largely empirical candidate screening and limited design guidelines, particularly in terms of saRNA targets. Unlike siRNAs, for which extensive sequence–activity relationships and computational design strategies exist, saRNA activity remains comparatively difficult to predict. Here, we assembled an updated and annotated database of publicly available saRNA sequences and paired it with an overview of the RNAa principles, the clinical and preclinical landscape of saRNA utility and current saRNA design guidelines. Finally, we outline opportunities for artificial intelligence-assisted rational design, highlighting how machine learning trained on curated saRNA datasets could accelerate discovery of novel saRNA candidates. Consolidation of saRNA records into a unified resource will hopefully enable systematic analysis of sequence features associated with activity and lay a foundation for moving saRNA design from largely empirical screening toward more rational design.

## 1. Introduction

We are entering an era of precision medicine in which artificial intelligence–enabled molecular design of biologics enables the precise targeting of specific molecular processes, accelerating the translation of biomedical research into clinical applications. Although small molecules still constitute most approved therapeutics, the portfolio of biologics based on nucleic acids and proteins continues to expand, particularly in the field of cancer and rare genetic diseases, which often result from loss-of-function (LOF) or gain-of-function (GOF) mutations [1,2]. Therapeutic strategies aimed at addressing such diseases include gene replacement therapy, genome-editing technologies, and the regulation of gene expression using noncoding RNA-based therapeutics, with small interfering RNAs (siRNAs) and antisense oligonucleotides (ASOs) at the forefront of this approach [3]. The modular and programmable nature of RNA therapeutics enables the targeting of diverse molecular substrates, including DNA, RNA, and proteins, thereby expanding the range of therapeutically accessible targets beyond those amenable to conventional small molecules [4,5]. RNA therapeutics can achieve high target specificity, are generally well tolerated, and allow controllable modulation of gene expression, factors that have contributed to their success in clinical development and therapeutic application. Unlike DNA-based therapies, RNA therapeutics do not permanently alter the genome [6]. Their large-scale production is also generally less expensive and less complex than that of DNA- or protein-based therapeutics [4].

Clinical success of siRNAs and ASOs, however, has been largely confined to gene silencing and, in selected cases, functional restoration through modulation of mRNA splicing. By contrast, RNA-based approaches that directly activate endogenous gene expression without altering the target mRNA sequence remain scarce [4]. In two seminal studies, Li et al. [7] and Janowski et al. [8] independently demonstrated that synthetic short RNA duplexes targeting gene promoters could activate, rather than silence, gene transcription, a phenomenon termed RNA activation (RNAa). These synthetic molecules, known as small activating RNAs (saRNAs) or as antigene RNA (agRNA) [9], but not to be confused with self-amplifying RNA (also abbreviated as saRNA), induce a moderate increase in endogenous gene expression and have thus far been confirmed for a variety of additional genes (Table 1 and Table 2). Two decades after the discovery of RNAa, the therapeutic potential of saRNAs has yet to be fully realized. Despite their unique ability to activate endogenous gene expression, progress in the field has been relatively modest, with three candidates now in clinical evaluation, in contrast to the rapid clinical translation of siRNAs and ASOs [6]. This limited progress is attributable, in part, to challenges associated with delivery, stability and specificity, but can also be ascribed to the limited success of predictive design principles [10], meaning that saRNA discovery still relies largely on empirical screening of multiple candidate sequences. Due to the extensive patenting of saRNA technologies (Table S1), the advances in design strategies may remain proprietary; therefore, the publicly available literature may not fully reflect the maturity of the field. Nevertheless, compared with the elaborate design principles for structurally similar siRNAs [11], publicly available guidelines for saRNA design remain limited [12].

Here, we review RNAa mechanistic principles and applications, followed by an overview of current saRNA design guidelines, their challenges and opportunities for artificial intelligence-assisted saRNA design. Importantly, we present an updated, extensively annotated database containing a large share of the reported and publicly available saRNA sequences to date, providing a comprehensive resource for identifying design principles and aiding the development of next-generation saRNA therapeutics.

## 2. RNA Activation: Mechanistic and Structural Principles

### 2.1. RNA Activation Mechanism

**Figure 1:**
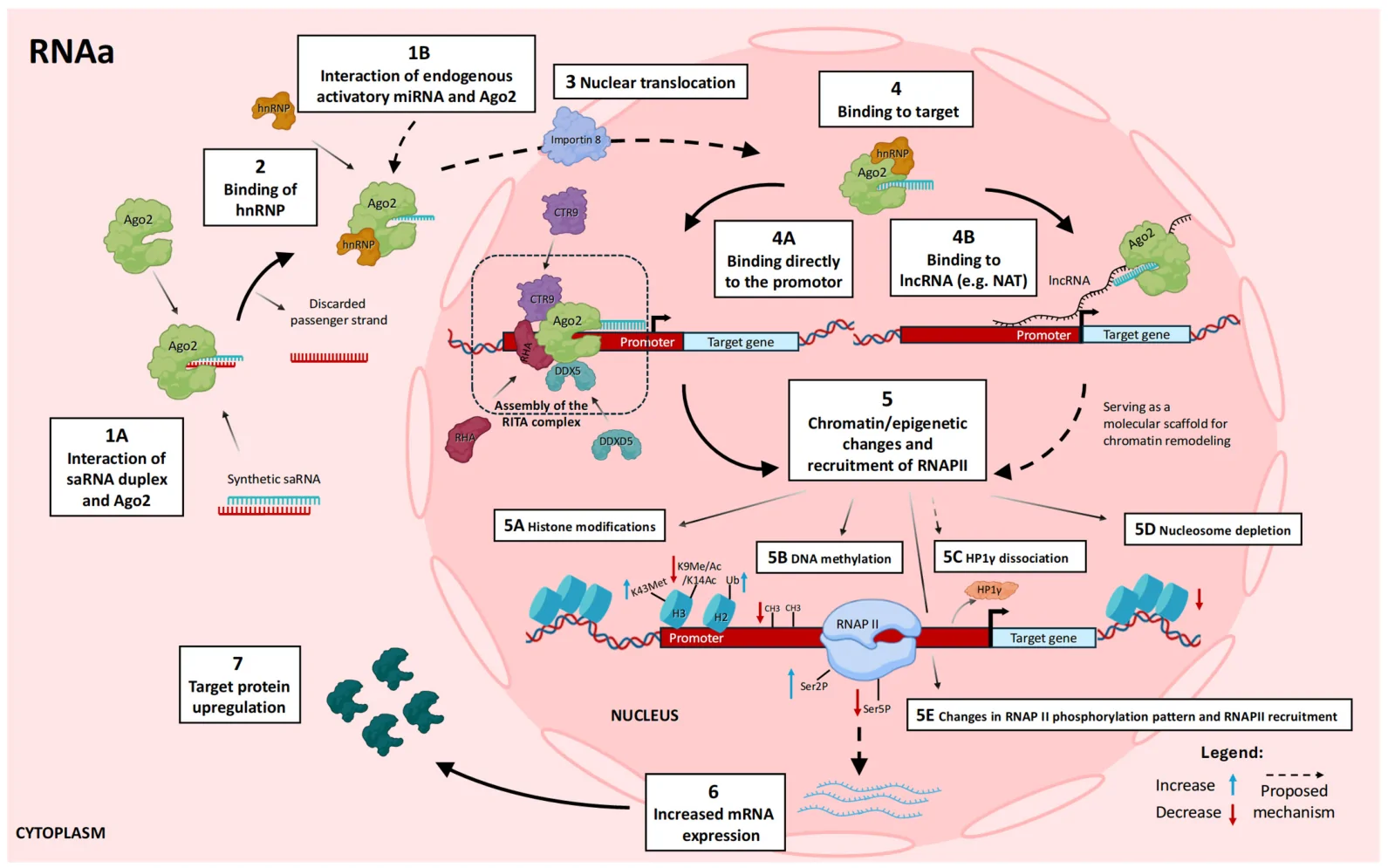
Molecular mechanisms of RNA activation (RNAa). In the cytoplasm, synthetic small activating RNA (saRNA) (**1A**) or endogenous activatory miRNA (**1B**) interacts with Ago2. saRNA interacts via its guide strand, while the passenger strand is discarded (**1A**). The saRNA-Ago2 complex is joined by several heterogeneous nuclear ribonucleoproteins (hnRNPs) (**2**) and then imported to the nucleus (**3**), where it can bind either a promoter (DNA) (**4A**) or long non-coding RNA (lncRNA), including natural antisense transcripts (NATs), transcribed from the promoter region (**4B**). Binding to the promoter is accompanied by the formation of an RNA-induced transcriptional activation (RITA) complex made of RNA helicase A (RHA), RNA polymerase-associated protein CTR9 homolog (CTR9), and DEAD-box helicase 5 (DDX5) (**4A**), while saRNA-NAT-Ago2 are proposed to serve as scaffolds (**4B**). Both pathways were linked with the recruitment of RNA polymerase II (RNAP II) and several chromatin/epigenetic changes of the target promoter (**5**), such as various histone modifications (**5A**), reduction of the DNA methylation of the distal promoter of the target gene (**5B**), decreased target promoter and heterochromatin-forming protein (HP1γ) association (**5C**), nucleosome repositioning and the formation of nucleosome-depleted regions (**5D**), or changes in the phosphorylation pattern of RNAP II (**5E**). These events likely lead to an increase in mRNA abundance (**6**) and protein upregulation (**7**).

The central component of the RNAa pathway is a short double-stranded RNA molecule, known as saRNA, which can upregulate gene expression through direct binding to the promoter region of a target gene or binding to the long noncoding RNA (lncRNA), most notably natural antisense transcripts (NAT), complementary to the target promoter (Figure 1). Naturally occurring activatory miRNAs [13] binding in the 5′ UTR region [14], promoter [15], on the TATA box [16], or the enhancers [17] are by some considered an endogenous RNAa modality, albeit mechanistically poorly understood (Figure 1) [18,19]. RNAa is evolutionarily conserved and has been identified across diverse groups of eukaryotes, ranging from nematodes to humans [20]. The exact molecular mechanism that underlies RNAa remains unknown and is, given the various binding targets, likely heterogeneous; however, saRNA activity does seem to universally depend on the involvement of the Argonaute (Ago) family proteins, most commonly Ago2 [7,21,22,23]. Ago2 is best known for its slicing role in the RNA-induced silencing complex (RISC), supporting RNA interference (RNAi) in the cytoplasm by siRNA-directed cleavage [24], and although functionally distinct, RNAa shares several structural features with the better-characterized RNAi pathway.

Upon entering the cytoplasm, the saRNA duplex is loaded by Ago2 and the passenger strand is discarded from the complex while the guide strand remains bound to Ago2 [21,22,23]. Following cytoplasmic loading of the saRNA duplex onto Ago2 and removal of the passenger strand, the guide strand and Ago2 complex is joined by several heterogeneous ribonucleoproteins (hnRNPs) [25,26] and then imported to the nucleus, likely via importin 8 [27]. The activation of the target gene occurs through a mechanism that has not yet been fully elucidated. Several studies demonstrated that in the nucleus, the saRNA-Ago2 complex can either directly bind DNA (the promoter region of the target gene) [21,22,27,28] or RNA (NAT) [29,30,31].

Binding of the saRNA-Ago2 complex to the target gene promoter is reported to occur similarly to miRNA binding to mRNA targets, in a seed-dependent manner [32]. It proceeds to induce assembly of the RNA-induced transcriptional activation (RITA) complex in the nucleus. RITA complex consists of RNA helicase A (RHA), RNA polymerase-associated protein CTR9 homolog (CTR9), and DEAD-box helicase 5 (DDX5), which interact with RNA polymerase II (RNAP II) to induce initiation of transcription and productive elongation [21,22,25,28]. The saRNA-Ago2 complex interaction with lncRNAs such as NATs is also reported to increase the recruitment of RNAP II to the promoter [28,29,30]. NATs may tether to the promoter region and serve as a scaffold for the saRNA-Ago2 complex [33], likely to anchor it in the vicinity of the target promoter. RNAa is unlikely to be the consequence of Ago2-mediated lncRNA knockdown as the lncRNA is reported to remain uncleaved, suggesting dispensability of Ago2 slicing properties [33]. In terms of miRNA involvement, there are many unknowns; however, it is tempting to speculate that they act as endogenous mimetics of the saRNA guide strand.

These events ultimately lead to RNAP II recruitment and various changes in the chromatin and epigenetic regulation [22,34]; however, the sequence of these events is not clear. RNAa induces nucleosome repositioning and the formation of nucleosome-depleted regions at TATA boxes, CpG islands, proximal enhancers, and proximal promoters [35]. The interaction with the target has been reported to increase the recruitment of RNA polymerase II (RNAP II) [21,22,25,28,29,30,31], as well as changes in the phosphorylation pattern of RNAP II [22]. An enrichment of Ser2P and depletion of Ser5P in RNAP II have been shown to stimulate both initiation and productive elongation [22]. Apart from the posttranslational modification of RNAP II, RNAa also influences histone modifications with reduction in levels of H3 lysine-9 (H3K9) dimethylation [7], H3K9 and H3K14 acetylation, and increased di/trimethylation at histone H3K4 and mono-ubiquitination of H2B [34]. As has been recently demonstrated, RNAa can also reduce the DNA methylation levels of the distal promoter of the target gene [36]. Additionally, Schwartz et al., 2008 observed reduced levels of association of the target promoter and a heterochromatin-forming protein (HP1γ) [29]. Together, these changes lead to higher transcriptional levels and an increase in mRNA abundance.

### 2.2. Composition of saRNA

**Figure 2:**
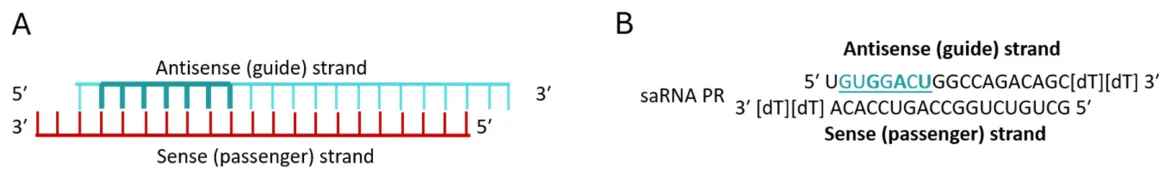
Schematic of the saRNA composition. (**A**) saRNA exists as a duplex of an antisense (guide) strand and a sense (passenger) strand. The antisense/guide strand binds to Ago2 and is complementary to the target, while the sense/passenger strand is removed from the Ago2 complex. (**B**) Located on the 5′ end of the guide/antisense strand is the seed region (bold/blue), which is responsible for target recognition and highly sensitive to mutations.

saRNA typically comprises a duplex of 19-nucleotide strands with two-nucleotide deoxythymidine (dTdT) 3′ overhangs on both strands [7,8] (Figure 2). It consists of a guide (antisense) strand, which is the strand that is loaded by Ago2 and is complementary to the target, and a passenger (sense) strand. Selection of the guide strand follows the thermodynamic asymmetry rule: the strand whose 5′-end exhibits lower thermodynamic stability is preferentially loaded as the guide strand, while the opposing strand is displaced and discarded as the passenger [22,23]. When binding the promoter, target recognition is primarily determined by the seed region, encompassing nucleotides 2–8 at the 5′ end of the guide strand [32]. Consequently, mutations within this region, particularly nucleotides in positions 4 and 6 on the guide strand, markedly affect saRNA activity, whereas mutations outside the seed region generally have little impact on its efficacy [32].

### 2.3. Characteristics of RNAa

A notable RNAa feature in comparison to other therapeutic strategies is that its genes are upregulated to moderate levels, often yielding only 2–3-fold upregulation of gene expression, though higher activation has been reported in some contexts [37] (Table 1). This may reflect the reliance of RNAa on the cell’s endogenous transcriptional machinery and the regulatory constraints imposed by the genomic and epigenetic context of the target locus (basal transcriptional activity, promoter accessibility, chromatin state, and the availability of endogenous transcriptional regulators). While this may limit the maximal level of gene induction, it may also provide a more physiologically regulated pattern of gene expression.

Furthermore, saRNA function appears to be cell-type specific [8]. *p21* promoter-targeting saRNA candidate dsP21-322, for example, exhibits variable activity when tested in different cell types (Table 1). This can be partly attributed to cell-specific transcriptional activity, promoter accessibility governed by DNA methylation status and chromatin state, and nucleosome positioning, as well as availability of RNAa-associated regulatory factors between cell types. Current evidence suggests that saRNA-mediated gene activation is most effective at transcriptionally competent loci that retain basal promoter activity but are also not highly expressed [10]. Genes that are epigenetically silenced or embedded within inaccessible chromatin are generally poor targets for saRNA-mediated activation, while overexpressed genes tend to have their endogenous transcription already maximized [7,10].

A unique feature of RNAa is also delayed onset of gene activation in comparison to siRNA. While siRNA-mediated effects can be seen after 6 h, maximal activation by RNAa occurs within a window of ~72–96 h, which was ascribed to additional rate-limiting steps in comparison to the RNAi mechanism, including nuclear context and effects on chromatin structure [10,23,28]. Some studies also report a prolonged effect lasting for 2 weeks [8].

**Table 1:** Reported activity of *p21*-targeting saRNA in different cell types. Effect reported refers to the fold response of *p21* mRNA expression in saRNA-treated cells in comparison to untreated control, as determined by qPCR.

saRNA Name in Reference	Concentration	Treatment Duration	Cell Type	Effect Reported	Reference
dsP21-322	50 nM	72 h following Lipofectamine 2000 transfection	PC-3	6.5	[38]
			Hep3B	3.8	
			HeLa	5	
			HEK293T	3.8	
			ACHN	2.5	
			T24	4.3	
			U2-OS	4.1	

## 3. Harnessing RNAa for Therapeutic Intervention

RNAa has been explored across a range of applications, including gene therapy, gene function studies, stem cell research, and cell reprogramming [39,40,41]. Three publicly disclosed saRNA therapeutics have advanced to clinical evaluation, two in oncology and one for neuromuscular disorder (Table 2 and Table S2). Preclinical saRNA exploration extends to a broad spectrum of diseases, including metabolic diseases, urinary tract disorders, reperfusion injury, blood disorders, ophthalmological diseases, lung diseases, neuromuscular disorders, haploinsufficiency-related conditions, tissue regeneration, and dermatological applications, which are listed in Table 3 together with the target genes and references. The clinical and preclinical landscape described below reflects publicly available information as of August 2026. Here, we discuss the most relevant or recent therapeutic applications in greater detail, while additional applications are covered in other comprehensive reviews [19,34,37,42,43,44,45,46].

### 3.1. Clinical Translation of saRNA

The first, MTL-CEBPA, developed by MiNA Therapeutics, entered Phase I clinical trials in 2016 for patients with advanced hepatocellular carcinoma [47,48]. MTL-CEBPA activates the *CEBPA* gene, encoding the transcription factor CCAAT/enhancer-binding protein alpha (C/EBPα), a master regulator of hepatocyte differentiation, metabolism, and immune function. The saRNA incorporates multiple 2′-O-methyl modifications to reduce innate immune activation and is encapsulated in SMARTICLES^®^ amphoteric liposomal nanoparticles for liver-targeted delivery. Preclinical studies demonstrated that MTL-CEBPA-mediated activation of endogenous *CEBPA* expression improved liver function parameters, reversed disease-associated changes in fibrosis and steatosis models, and reduced tumor burden in cirrhotic hepatocellular carcinoma models [49]. Phase I studies showed that MTL-CEBPA was generally well tolerated, could be safely combined with sorafenib, and induced reductions in circulating myeloid-derived suppressor cells, supporting both direct antitumor and immune-modulatory effects [47]. A subsequent non-comparative, open-label Phase I study combining MTL-CEBPA with pembrolizumab in 50 patients with advanced solid tumors confirmed this favorable safety profile and provided preliminary evidence of clinical activity, including 4 confirmed partial responses among 40 evaluable patients [50]. As a single-arm early-phase study, these results should be interpreted as preliminary.

More recently, in 2024, RAG-01, developed by Ractigen Therapeutics, entered Phase I clinical evaluation for the treatment of non-muscle-invasive bladder cancer [51]. RAG-01 activates the *p21* (*CDKN1A*) gene, encoding the cyclin-dependent kinase inhibitor p21, a key regulator of cell-cycle progression and genomic stability [52]. Preclinical studies demonstrated induction of p21 expression and inhibition of tumor cell proliferation, providing the rationale for clinical translation [53,54]. The ongoing Phase I trial evaluates the safety, tolerability, pharmacokinetics, and preliminary antitumor activity of RAG-01, which has also received NMPA/CDE clearance to initiate a Phase II trial in China in March 2026.

Ractigen Therapeutics has also advanced RAG-18, an saRNA targeting utrophin (*UTRN*) for Duchenne muscular dystrophy, with the first patient dosed in an investigator-initiated trial in December 2025 [55]. Utrophin is a functional paralogue of dystrophin, and its upregulation can compensate for primary dystrophin defects such as Duchenne muscular dystrophy and Becker muscular dystrophy. In preclinical studies, RAG-18 effectively mitigated muscle damage and thus received a Rare Pediatric Disease Designation from the U.S. Food and Drug Administration (FDA) [56], which was followed by the granting of Orphan Drug Designation (ODD) in 2024 [57].

**Table 2:** Current overview of clinically relevant saRNA.

Drug	Target and Targeting Modality	Sequence (5′ → 3′)	Chemical Modifications	Delivery and Route
MTL-CEBPA	*CEBPA*, Promoter targeting	Sense: mGmCGmGUCAUUmGUCAmCUGGUCmUmU Antisense: GACCAGUGACAAUGACCGCmUmU	2′-O-methyl modification, 5′ inverted abasic cap (sense strand)	SMARTICLES^®^ liposomes
RAG-01	*p21*, Promoter targeting	Not publicly disclosed	Partial chemical modification (specific pattern not disclosed); C5X5 lipid conjugate	LiCO™; intravesical
RAG-18	*UTRN*, Promoter targeting	Not publicly disclosed	LiCO™ lipid conjugate (details not disclosed)	LiCO™; IV/subcutaneous

### 3.2. Preclinical saRNA Landscape

#### 3.2.1. Ophthalmological Diseases

Targeting *p21* has shown promise not only in oncology but also in proliferative vitreoretinopathy, where standard surgical approaches, although producing satisfactory results, are commonly accompanied by complications and/or reduced visual acuity [58]. In a recent study by Zhang et al. (2023), saRNA-activated p21 demonstrated anti-proliferative and anti-migratory effects, inhibiting the progression of proliferative vitreoretinopathy in a rabbit model [58]. Following up, RAG-1C (Ractigen Therapeutics), an investigational saRNA therapeutic targeting p21 for proliferative vitreoretinopathy, received Investigational New Drug (IND) clearance from China’s National Medical Products Administration (NMPA/CDE) in March 2025 and from the U.S. Food and Drug Administration (FDA) in July 2026, authorizing initiation of a first-in-human Phase I clinical trial [59].

#### 3.2.2. Oncology

Numerous saRNAs targeting other cancer-related genes are being developed, making oncology the most explored field for therapeutic applications of RNAa. RNAa has been proposed as either a standalone cancer treatment or a combinational therapy with already approved anticancer drugs. As the latter, RNAa has been used to enhance the antitumor activity of some already established anticancer treatments such as cisplatin [60], antibodies [61], and chimeric antigen receptor (CAR)-T cell therapy [62]. While CAR-T therapy has achieved impressive results in the treatment of hematological malignancies, its application to solid tumors has been less successful due to a uniquely hostile tumor microenvironment that impairs T cell function. To address one of the most critical factors, hypoxia, Huang et al. (2026) applied saRNA targeting HIF-1α to CAR-T cells using the piggyBac transposon system [62]. HIF-1α upregulation boosted initial expansion and enriched the Tcm phenotype. Additionally, it enhanced resistance to exhaustion in multiple CAR-T designs targeting different ligands, which resulted in significantly reduced tumor burden and prolonged survival in the SKBR3 xenograft mouse model. RNAa thus presents a promising approach for overcoming metabolic barriers that limit the efficacy of CAR-T cells in solid tumor immunotherapy.

Additionally, RNAa has shown potential in combating drug resistance in various cancers, which limits the duration of response to treatment, hinders long-term survival in cancer patients, and presents a significant challenge for anticancer therapy [63,64]. Recent studies of RNAa in oncology also focus on innovative delivery systems that improve saRNA stability and enhance tumor targeting. In a study by Wang et al. (2024), saRNA targeting *PTPRO* was encapsulated in mesoporous silica-based nanoparticles coupled with trastuzumab (saPTPRO-MSNP-TZM), which exhibited preferential tumor localization and increased cellular uptake of the drug, leading to higher upregulation of *PTPRO* [63].

Furthermore, Yu et al. (2026) designed saRNAs for p38 and TFEB upregulation, encapsulated within a metal-organic framework (MOF)-based delivery system and cloaked with hybrid membranes composed of synthetic lipids and exosome-derived vesicles for the treatment of lung cancer [65]. A key component of the tumor environment in lung cancer is tumor-associated macrophages, consisting of two primary subtypes: M1 macrophages with antitumor activities and M2 macrophages that facilitate tumor progression. saRNA encapsulated in engineered nanoparticles, further functionalized with the macrophage-targeting peptide CRV, reprogrammed the macrophage phenotype from M2 to M1 and enhanced the antitumor immune response, resulting in significant tumor suppression compared with controls, with no notable weight loss or major organ toxicity observed in LLC tumor-bearing mice [65].

#### 3.2.3. Neuromuscular Disorders

Apart from primary dystrophin defects, spinal muscular atrophy (SMA), which is caused by mutations in the Survival of Motor Neuron 1 (*SMN1*), is another hereditary neuromuscular disorder that could be addressed by RNAa. While there is no cure for SMA, some strategies for symptom management and complication prevention include upregulating the levels of the functional *SMN2* gene that encodes the same protein as *SMN1*; however, due to a mutation in *SMN2* that affects its splicing, only a small amount of *SMN2* produces a functional full-length protein. An approved antisense oligonucleotide, Spinraza^®^ (nusinersen), modulates the splicing of *SMN2* pre-mRNA, but its efficacy is limited by the available amount of *SMN2* pre-mRNA. RNAa could avoid this limitation. In a 2019 patent by Li et al., they proposed using saRNA to upregulate *SMN2* and reported increased expression of full-length SMN2 protein in cell-based assays [66].

#### 3.2.4. Metabolic Disorders

Metabolic disorders are characterized by imbalanced energy homeostasis and are a leading cause of chronic illness worldwide [67]. They are diverse and affect multiple biochemical pathways that can potentially be targeted by RNAa. Matsui et al. achieved an increase in surface LDLR expression via RNAa comparable to that induced by the approved cholesterol-lowering drug lovastatin in HepG2 cells, providing encouraging results for further development of RNAa for *LDLR* upregulation to combat hypercholesterolemia, a major modifiable risk factor for atherosclerotic cardiovascular disease [31]. Another promising target for the treatment of metabolic diseases is *SIRT1* [68]. In a recent study by Cai et al. (2024), saRNA targeting *SIRT1*, delivered by a tetrahedral framework nucleic acid, reconstructed the immune microenvironment in the bone tissue of a mouse model of diabetic osteoporosis, a common but often overlooked secondary complication of diabetes mellitus [67]. To address another complication of diabetes mellitus that affects 25–75% of diabetic men—erectile dysfunction, Wang et al. (2013) designed saRNA for upregulation of inducible nitric oxide synthase (iNOS) that activated the NO/intracellular cyclic GMP pathway and improved the erectile function of diabetic rats [69].

Ractigen Therapeutics developed saRNA targeting the hydroxymethylbilane synthase (*HMBS*) gene, deficiency of which causes the autosomal dominant genetic disease acute intermittent porphyria. SaRNA targeting *HMBS* successfully activated both mRNA and protein expression of the affected gene in GM01625 cells from patients with acute intermittent porphyria and demonstrated that, in addition to the previously described pathologies in lipid and glucose metabolism, RNAa can also address disorders related to disruptions in the heme biosynthetic pathway, such as hereditary porphyria [70].

#### 3.2.5. Kidney/Urinary Tract Diseases

In 2014, Yang et al. demonstrated that *TRPV5*, a gene encoding a calcium-selective cation channel that regulates cellular calcium transport in the kidneys, can be activated by saRNA and proposed its use in the treatment of Ca^2+^-balance-related diseases [71]. Zeng et al. (2018) showed that upregulation of *TRPV5* by saRNA reduces renal calcium oxalate deposition in vivo, one of the most common causes of kidney stones, by increasing the reabsorption of calcium ions in the kidneys and reducing calcium levels in the urine, thereby decreasing kidney stone formation [72]. Other promising targets for saRNA-mediated upregulation to address urinary tract pathologies are *TREK-1* [73] and *MyoD* [74], for the treatment of detrusor overactivity and stress urinary incontinence, respectively.

#### 3.2.6. Lung Injuries

Apart from the treatment of advanced hepatocellular carcinoma, upregulation of CEBPA has been shown to be applicable also for respiratory disease. Zhang et al. (2024) explored the anti-inflammatory effect of *CEBPA* for the treatment of acute lung injury/acute respiratory distress syndrome, disorders characterized by uncontrollable inflammation in the lungs [75]. *CEBPA*-saRNA, complexed with histone 1 and coated in a neutrophil membrane, exhibited effective M1/M2 macrophage polarization and a significant anti-inflammatory effect in an LPS-induced acute lung injury mouse model.

#### 3.2.7. Reperfusion Disorders

*VEGF* gene for vascular endothelial growth factor, *CDH1* for E-cadherin and *p21*, were among the first genes found to be upregulated by RNAa [7]. As a key mediator of angiogenesis and vascular permeability, RNAa-activated VEGF improved blood flow in mice with ischemic hindlimbs, reduced infarct size in a myocardial infarction model [76], and prompted changes in extravillous trophoblast cells that are beneficial for preeclampsia recovery [77]. Recent research on cardiac contractile function and pathological post-myocardial infarction remodeling has identified βII spectrin (*SPTBN2*) as another potential therapeutic target for myocardial infarction [78]. Activation of *SPTBN2* via saRNA in the hearts of mice in a myocardial I/R injury model alleviated I/R-induced cardiac contractile dysfunction, cardiomyocyte death, and mitochondrial damage.

Apart from ischemic diseases, RNAa can also be used as an intervention for hemorrhage disorders. Intracerebral hemorrhage is a type of stroke caused by bleeding within the brain parenchyma due to non-traumatic factors, with a high fatality rate (45.6%). A very recent study by Xu et al. (2026) identified *ATF3* as a therapeutic target for intracerebral hemorrhage and engineered a biomimetic, brain-targeted nanoplatform (L57-NExos@saRNA) designed for the precise cerebral delivery of therapeutic saRNA [79]. Upregulation of *ATF3* with L57-NExos@saRNA in a mouse model of intracerebral hemorrhage led to improved neurological impairment according to the Garcia test, restored turning behavior, accelerated hematoma resolution, and reduced brain water content; it also preserved neuronal morphology with reduced inflammation and decreased secondary neurotoxicity by facilitating iron clearance. Collectively, the study demonstrated that L57-NExos@saRNA promoted both functional and cognitive recovery of affected mice.

#### 3.2.8. Blood Disorders

In a 2020 patent, Li et al. proposed using saRNA targeting thrombopoietin (*THPO*) for the treatment of thrombocytopenia [80]. Due to its thrombopoietic effects, THPO has been widely explored as a therapeutic agent for the treatment of thrombocytopenia, with early clinical trials of recombinant human thrombopoietin showing promising results. However, subsequent studies identified neutralizing antibodies against both the recombinant and endogenous THPO, resulting in persistent thrombocytopenia. An approach that upregulates endogenous *THPO* via RNAa and thus avoids this problem presents a clear advantage over the more traditional strategy using recombinant THPO.

#### 3.2.9. Haploinsufficiency and Dosage Sensitivity-Related Disorders

The modest upregulation exhibited by saRNAs, their titratability, and their targeting of endogenous gene regulation make them particularly promising for rare monogenic disorders caused by loss-of-function mutations in haplo-insufficient genes, especially those with a narrow dosage sensitivity window [81]. Mutations affecting one of the two alleles of a haplo-insufficient gene lead to loss of function of that allele, and because the remaining wild-type allele is insufficient to produce the quantity of protein required to support normal cellular structure and signaling, the resulting reduction in gene output leads to disease. In principle, carefully increasing expression from the intact allele toward physiological levels could compensate for decreased gene output, with saRNAs offering a potential means to achieve such controlled endogenous upregulation [82].

As proof of concept, Fimiani et al. demonstrated that promoter-targeting saRNAs can upregulate endogenous Foxg1 expression in the mouse neocortex, providing evidence that RNA activation may represent a therapeutic strategy for haplo-insufficient neurodevelopmental disorders [82]. Additionally, Bezalel, Darr, and Perets designed a series of saRNAs targeting the *SHANK3* promoter for the treatment of Phelan–McDermid syndrome and other intellectual disabilities associated with *SHANK3* haploinsufficiency [83]. In human KG-1 cells, the lead molecule upregulated *SHANK3* mRNA by up to 2.1-fold at 72 h post-transfection, exceeding the 1.4-fold induction achieved by lithium as the positive control [83]. These findings underline RNAa as a promising alternative to gene therapy for restoring expression of the wild-type allele in haploinsufficiency-related disorders, though this potential has yet to be fully realized.

**Table 3:** Pathologies and associated saRNA targets. For saRNA in clinical trials, gene targets are in **bold**. SaRNA targets are marked with ˝†˝ if tested in vitro and ˝*˝ if tested in vivo.

Pathology		Target Gene	In Vitro Studies (†)	In Vivo Studies (*)
Cancer	Prostate cancer	*p21* †/*	[7,23,26]	[84]
		*p53* †	[85]	
		*NKX3-1* *		[86,87]
		*KLF4* †/*	[88]	[87]
		*PAWR* †	[89]	
		*INTS6* †/*	[90]	[91]
		*Notch1* *		[92]
		*KLK1* †	[27]	
	Bladder cancer	***p21*** †/*	[53,93]	[54,94]
		*p53* *		[95]
		*PAWR* †	[60,89]	
		*CDH1* †	[96,97]	
	Renal cell carcinoma	*VHL* †	[98]	
		*DIRAS1* *		[99]
	Breast cancer	*HIC1* †	[100,101]	
		*MAS1* *		[102]
		*PTPRO* *		[63]
	Ovarian cancer	*MAS1* *		[102]
	Lung cancer	*PTEN* †	[103]	
		*VDUP1* *		[104]
		*RUNX3* †	[105]	
		*p38* *		[65]
		*TFEB* *		[65]
	Liver cancer	***CEBPA*** *		[25,47,49,50,106]
		*WT1* †	[107]	
		*LHPP* *		[61]
		*p21* †	[108]	
	Colorectal cancer	*KLF4* †	[109]	
		*VMP1* †	[110]	
		*p21* †/*	[38,111]	[112]
	Gastric cancer	*p53* †	[113]	
		*VEZT* †	[114]	
		*HIC1* †	[115]	
	Pancreatic cancer	*CEBPA* *		[116]
		*MAS1* *		[102]
	Thyroid cancer	*NIS* *		[117]
	Pheochromocytoma	*p53* *		[118]
	Chronic myeloid Leukemia	*miR-181a* *		[119]
		*CDH13* *		[64]
	Glioma	*p21* †	[120]	
		*ALOX15* *		[121]
Metabolic disorders	Hypercholesterolemia	*LDLR* †	[31,122]	
	Secondary complications of diabetes mellitus	*iNOS* *		[69]
		*SIRT1* *		[68]
	Metabolic syndrome	*SIRT1* *		[123]
	Acute intermittent porphyria	*HMBS* †	[70]	
Urinary tract disorders	Kidney stones	*TRPV5* †/*	[71]	[72]
	Stress urinary incontinence	*MYOD* †	[74]	
	Detrusor overactivity	*TREK-1* †		[73]
Reperfusion disorders	Myocardial infarction	*VEGF* †	[76]	
		*SPTBN2* *		[78]
	Preeclampsia	*VEGF* †	[77]	
	Intracerebral hemorrhage	*ATF3* *		[79]
Blood disorders	Thrombocytopenia	*THPO* *		[80]
Neuromuscular disease	Spinal muscular atrophy	*SMN2* *		[66]
	Dystrophin-deficient-related disorders (DMD, BMD)	***UTRN*** †	[55]	
Respiratory disorders	Acute lung injury/acute respiratory distress syndrome (ALI/ARDS)	*CEBPA* *		[75]
Ophthalmological disorders	Proliferative vitreoretinopathy	*p21* *		[58]
Haploinsufficiency related disorders	FOXG1-related haploinsufficiency	*FOXG1* *		[82]
	Intellectual disabilities associated with SHANK haploinsufficiency	*SHANK3* †	[83]	
Dermal conditions	Skin care	*MFAP2* †	[124]	
		*COL1A1* †		
		*COL1A2* †		
		*COL3A1* †		
		*AQP9* †		
		*ELN* †		
Tissue Regeneration		*ATOH1* †	[125]	

## 4. Barriers to Broader Applicability of RNAa

Similar to other RNA therapeutics, saRNAs can face several biological and technological barriers that limit their clinical application, including inefficient delivery, limited stability, and off-target effects [46,126].

### 4.1. Delivery

Owing to their size, hydrophilicity, and negative charge, saRNAs also have limited penetration across the cell membrane and are primarily internalized by endocytosis [43]. They are also prone to nuclease degradation and renal clearance. Several delivery systems, including lipid-based nanocarriers, dendrimers, saRNA conjugates with aptamers, nanostructures, and biomimetic exosomes, have so far been proposed to protect the saRNA duplex, facilitate cellular uptake, or promote endosomal escape for clinical applications [43] (Table 4).

LNPs and related lipid-based nanocarriers are among the most advanced delivery vehicles for RNA therapeutics, including saRNA. Conventional LNPs typically comprise an ionizable lipid, helper phospholipid, cholesterol, and PEG-lipid, which together facilitate RNA encapsulation, particle stability, cellular uptake, and, depending on the formulation, endosomal escape and intracellular release. Lipidoid nanoparticles carrying p21-saRNA induced *p21* expression and inhibited prostate tumor growth [84], while LNP-mediated intravesical delivery of modified p21-saRNA suppressed orthotopic bladder tumors [54]. Related lipopolyplexes, which combine a polymer–saRNA complex with a lipid shell, have also been developed for saRNA delivery. In colorectal cancer, a lipopolyplex comprising a PEI/p21-saRNA polyplex core and a hyaluronan-modulated lipid shell improved local retention and promoted CD44-mediated tumor targeting and cellular uptake [127]. Another lipid-based platform for saRNA delivery is SMARTICLES^®^, an amphoteric liposomal system composed of both anionic and cationic lipid components, including phosphatidylcholine, DOTAP, dimyristoylglycerol hemisuccinate, and cholesterol [49]. The combination of oppositely charged lipids gives SMARTICLES^®^ a pH-dependent surface charge, which can facilitate cellular uptake under acidic conditions and promote pH-triggered endosomal escape. SMARTICLES^®^ have been used to deliver *CEBPA*-targeting saRNA (MTL-CEBPA), enabling *CEBPA* gene activation and producing therapeutic effects in preclinical models of hepatocellular carcinoma and liver diseases.

Moreover, DNA-based nanostructures, particularly tetrahedral framework nucleic acids (tFNAs), have emerged as versatile scaffolds for saRNA delivery. Their sequence-defined architecture can protect saRNA cargo and facilitate cellular uptake, while their modular design allows saRNA to be incorporated or hybridized with the DNA framework and enables additional functionalization with targeting ligands or stimuli-responsive elements. These features provide opportunities for efficient delivery, cell-specific targeting, and controlled intracellular release of gene-activating saRNAs. For example, a p21-saRNA-loaded tFNA enabled RNase H-responsive intracellular release of saRNA, whereas a CEBPA-saRNA-loaded tFNA was functionalized with a transferrin receptor aptamer (tTR14) for targeted delivery to pancreatic cancer cells [63]. Similarly, a SIRT1-saRNA-functionalized tetrahedral DNA nanostructure promoted *SIRT1* gene activation and attenuation of bladder fibrosis [128].

Beyond DNA nanostructures, poly-lysine-modified H-apoferritin nanocages have also been developed for saRNA delivery, where the poly-lysine modification promoted lysosomal escape through a proton-sponge effect and pH-induced degradation of the nanocage enabled cytoplasmic saRNA release, resulting in *SIRT1* activation and improved cartilage protection in osteoarthritis [129].

Dendrimers are highly branched polymeric nanocarriers whose surface functional groups can interact electrostatically with the negatively charged phosphate backbone of RNA, enabling the formation of nanoscale RNA–dendrimer complexes. In the study by Xiong et al., an amphiphilic dendrimer (AD) was used to deliver *MAS1*-targeting saRNA [102]. The resulting complexes protected saRNA from RNase and serum degradation, shielded its negative charge, and promoted cellular uptake, forming small spherical complexes of approximately 40 nm. Importantly, the system did not incorporate a separate targeting ligand; rather, therapeutic specificity was provided by the sequence-specific *MAS1*-targeting saRNA, which increased *MAS1* expression and suppressed tumor progression in multiple cancer models.

Furthermore, saRNA can be conjugated to aptamers, short oligonucleotides that bind specific molecular targets with high affinity and specificity, enabling cell-selective targeting and uptake through interactions with cell-surface molecules [116].

Despite the development of diverse delivery platforms, efficient saRNA delivery remains challenging, particularly for extrahepatic tissues [43,130]. Following systemic administration, many nanocarriers exhibit preferential hepatic accumulation, limiting delivery to non-hepatic target organs and specific cell populations. Moreover, cellular uptake does not necessarily result in productive delivery, as endosomal entrapment remains a major rate-limiting barrier, with only a small fraction of internalized RNA reaching the cytoplasm. Thus, further advances in saRNA delivery will require improved tissue- and cell-specific targeting as well as efficient and nontoxic mechanisms for endosomal escape.

### 4.2. Stability

RNA molecules are highly susceptible to RNase-mediated degradation, limiting their stability, circulation time, and bioavailability following systemic administration [43,126]. Chemical modifications can improve nuclease resistance, serum stability, and pharmacokinetic properties and reduce immunostimulatory effects; however, excessive or inappropriate modification intended to enhance protection can alter the structural and thermodynamic properties of saRNA, including duplex architecture, GC content, and thermodynamic asymmetry, and may consequently impair biological activity. A wide range of individual chemical modifications has been reported to be tolerated by saRNA, yet heavy modification of both strands—particularly 2′-O-methyl substitution of the guide strand—can abolish gene activation [131]. Recent work demonstrated that rationally combining thermally destabilizing and affinity-enhancing modifications can simultaneously improve duplex properties, nuclease stability, and gene activation [132]. Thus, effective saRNA design requires a balance between protection from degradation and preservation of the structural and thermodynamic features required for gene activation. saRNA design considerations in terms of chemical modifications are discussed in Section 5.

**Table 4:** saRNA delivery and administration routes proposed for different disease contexts.

Targeted Disease	Target Gene	Delivery Technique	Administration Route	Reference
Prostate cancer	*p21*	Lipidoid nanoparticles (LNP)	Intratumoral	[84]
Bladder cancer		Lipid nanoparticles (LNP)	Intravesical	[54]
Colorectal cancer		Tumor-selective lipopolyplex	Rectal	[127]
Cancer (anti-tumor)		Bioswitchable tFNA (pH-responsive)	Systemic	[133]
Liver cancer (HCC)	*CEBPA*	SMARTicles (amphoteric liposomes)	Intravenous	[21]
Liver cancer (HCC, cirrhotic)				[49]
Advanced HCC				[47]
HCC + atezolizumab				[50]
Pancreatic cancer		CEBPA-saRNA-PDAC specific P19/P1 aptamer conjugate	Systemic	[116]
Pancreatic cancer (PDAC)		tFNA + TfR aptamer	Systemic	[134]
Metabolic syndrome	*SIRT1*	TfR aptamer-saRNA conjugate	Subcutaneous	[123]
Diabetic osteoporosis		tFNA (tetrahedral framework nucleic acid)		[68]
Bladder fibrosis		tFNA	Local	[128]
Osteoarthritis		Poly-lysine apoferritin nanocage + hydrogel	Intra-articular	[129]
Ovarian, breast, pancreatic cancer	*MAS1*	Amphiphilic dendrimer (AD)	Intraperitoneal subcutaneous	[102]
Breast cancer (HER2+)	*PTPRO*	Mesoporous silica NPs + trastuzumab (saPTPRO-MSNP-TZM)	Systemic	[63]
Intracerebral hemorrhage (ICH)	*ATF3*	Biomimetic exosomes (platelet-neutrophil hybrid membrane)	Systemic	[79]
Lung cancer	*p38/TFEB*	MOF-based hybrid membrane nanoparticles	Systemic	[65]

### 4.3. Off-Target Effects

Another observed saRNA issue—off-target effects [46,84,135]—can result from improper selection of the passenger strand in saRNA duplexes and consequently its interaction with non-specific transcripts or gene promoters complementary to the passenger strand [23]. Studies of structurally related siRNAs demonstrate that chemical modifications can reduce unintended sequence-dependent interactions [136]. In saRNA, modification of the passenger strand can be used to suppress unintended strand activity, as blocking the 5′ end of the passenger strand reduced its off-target activity [23]. However, such modifications do not eliminate the need for careful sequence and strand selection, as unintended interactions may still arise from guide sequence similarity to other genomic or transcriptomic targets. Thus, minimizing off-target effects requires a combination of rational target selection, appropriate strand design, and chemical modifications.

### 4.4. Design Challenges

General saRNA design guidelines exist and have improved candidate prioritization [10]; however, they remain insufficient for reliably distinguishing highly active saRNAs from ineffective candidates across different genomic loci. This is largely a consequence of incomplete understanding of the molecular mechanisms underlying RNAa as well as the likely mechanistic heterogeneity of RNAa.

Importantly, saRNA activity can be highly cell-type dependent [7], with the same candidate displaying robust activation in one cellular context but limited or no activity in another (Table 1). Consequently, current approaches still heavily rely on empirical screening of multiple candidate sequences [10].

The success of designed saRNA also strongly depends on the thermodynamic asymmetry of its ends. When thermodynamics favors the sense strand (i.e., the strand not complementary to the target), the saRNA is typically inactive, which is an important source of the empirical “hit-or-miss” design problems. Chemical modifications such as 5′-terminal mismatches can be used to engineer the desired asymmetry and enforce antisense-strand loading [22,23,132].

## 5. Designing saRNAs: Lessons from Empirical Studies

**Figure 3:**
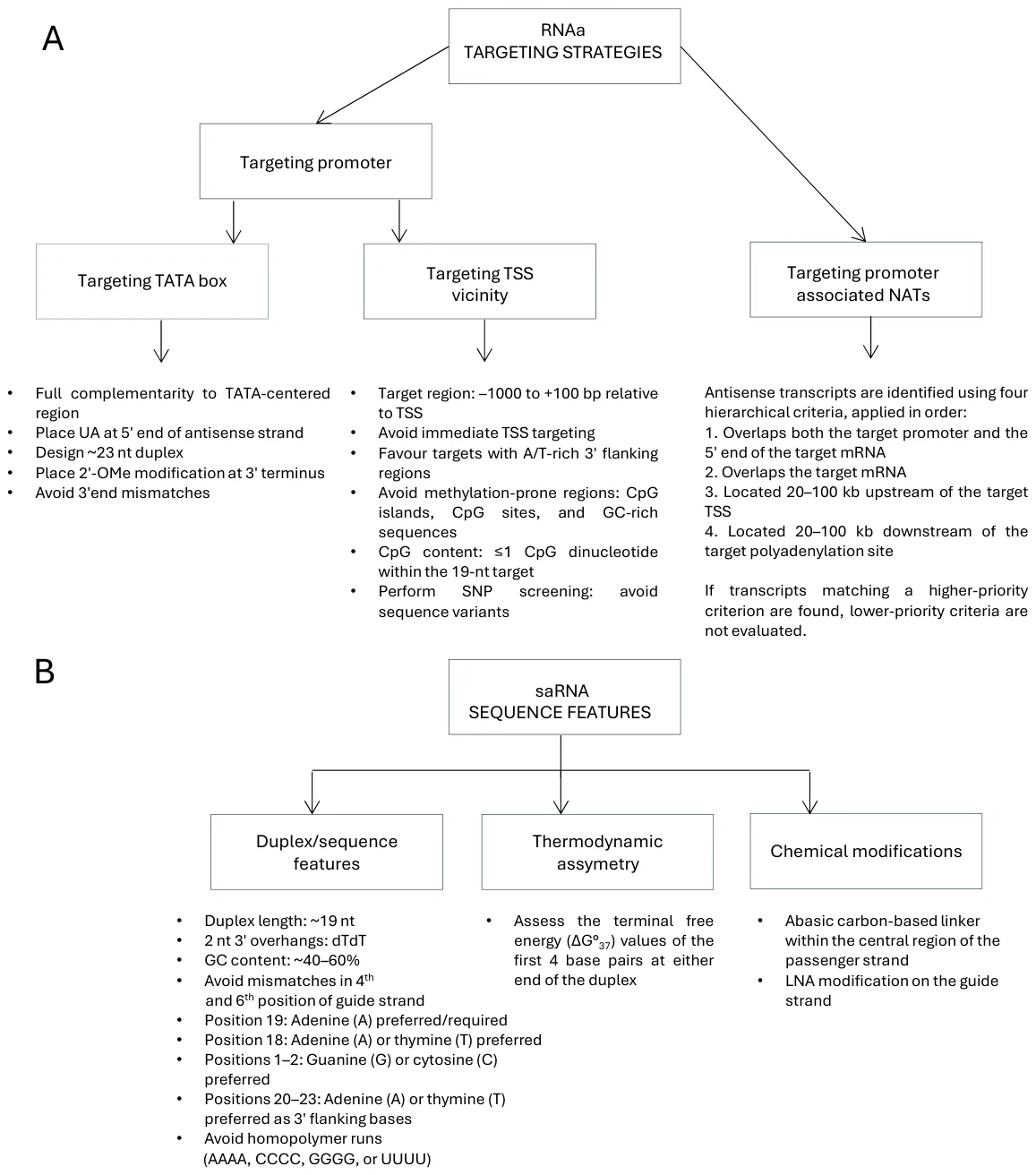
Schematic overview of reported RNAa targeting strategies (**A**) and saRNA design considerations (**B**).

Currently, publications by Wang et al. (2015) and Voutila et al. (2012) offer the most comprehensive, publicly available guidelines on saRNA design for promoter-targeting and NAT-targeting saRNA, respectively [10,41]. They are derived primarily from the empirical observations obtained from early RNAa studies and incorporate several principles, originally established for siRNA design. The design criteria for promoter targeting saRNAs reportedly yield active candidates in approximately 15–20% of designed sequences and are broadly divided into two categories: target promoter sequence properties and saRNA duplex sequence [10]. A summarized representation of saRNA targeting and design strategies is shown in Figure 3.

### 5.1. saRNA Sequence

The existing guidelines for saRNA duplex properties mostly focus on positioning, pairing, and content of particular nucleotides. saRNA should comprise a duplex with a strand length of approximately 19 nucleotides. It should include two-nucleotide overhangs on the 3′ side of both strands, moderate GC content, generally between 40% and 60%, and asymmetric thermodynamic stability between the two strands to promote guide strand selection and sequence features influencing Argonaute loading and intracellular processing [10,20]. The latter should be calculated as the terminal free energy (ΔG°_37_) of the first 4 base pairs at each end of the duplex as reported previously [137]. Additionally, removal of all candidates with homopolymeric motifs (aaaa, cccc, gggg, or uuuu) as well as di- or tri-nucleotide repeats is advised [10,41].

### 5.2. saRNA Chemical Modifications

Successful saRNA design may involve the introduction of chemical modifications to impose the thermal asymmetry required for preferential loading of the guide strand, improve in vivo saRNA stability, as well as reduce immunogenicity and off-target effects. Systematic assessment of how various chemical modifications affected saRNA performance and stability revealed that a strand-asymmetric modification rather than uniform positioning of modifications is preferential in terms of saRNA stability [132]. A thermally destabilizing abasic carbon-based linker positioned in the central region of the sense (passenger) strand, combined with affinity-enhancing LNA residues on the antisense (guide) strand, yielded the best balance of duplex melting temperature, nuclease stability, and gene activation [132]. The selection of chemical modifications requires consideration of their type, position, and strand specificity, as modifications that are well tolerated at one position or strand can substantially impair RNAa activity when introduced elsewhere [23,131].

### 5.3. saRNA Target Site Selection

#### 5.3.1. Promoter Targeting (Proximity to TSS) [10,20]

Functional saRNA targets are frequently identified within promoter regions located approximately −1000 to +100 bp relative to the transcription start site (TSS), with many active candidates reported within regions approximately −200 to −500 bp upstream of the TSS. Promoter regions immediately surrounding the TSS are often avoided because they frequently contain multiple transcription initiation sites and may have sequence characteristics that reduce predictability. However, a promoter’s position alone is insufficient to determine activity, and successful activation appears to depend on additional locus-specific factors. Identification of suitable target regions is typically assisted by genome annotation resources such as the UCSC Genome Browser and Ensembl, which provide information on gene structure, transcription start sites, transcript orientation, and alternative promoters. Additional genomic features, including RNA polymerase II occupancy and chromatin-associated marks, may provide further information regarding regulatory regions, although their predictive value for saRNA activity remains incompletely defined.

Once a target region is selected, the saRNA is designed against the sense DNA strand, with the complementary RNA strand serving as the guide strand. Several position-specific nucleotide preferences have been reported, including a requirement for adenine at position 19, a preference for adenine or thymine at position 18, guanine or cytosine at positions 1 and 2, and adenine or thymine at positions 20–23 flanking the 3′ end. Where possible, targets flanked by A/T-rich sequences on the 3′ side are favored, as these regions correlate with reduced nucleosome occupancy and improved promoter accessibility. Regions susceptible to DNA methylation—such as CpG islands, CpG sites, and GC-rich sequences—are avoided, as methylation has been shown to interfere with RNAa activity; targets should contain no more than one CpG dinucleotide within the 19-nucleotide window. Finally, selected targets should be screened for single-nucleotide polymorphisms, as sequence variation within the target site may compromise saRNA efficacy.

#### 5.3.2. Promoter Targeting (TATA Box)

In approximately 24% of human protein-coding genes, the core promoter contains a TATA box, an element that recruits the transcription pre-initiation complex [138]. An activating effect on the expression of interleukin-2 (IL-2) was observed with duplex RNA designed as siRNA when targeting the promoter region. Several rational design rules were suggested to design such activating siRNA, including complementarity to the TATA-box-centered region: usage of uracil and adenine (UA) as the first two bases of the antisense strand, length of 23 nucleotides, inclusion of 2′-O-Methyl (2′-OMe) modification at the 3′ terminus of the antisense strand, as it extended activation to more than 4 days and avoidance of mismatches at the 3′ end of the antisense strand in addition to seed region mismatches [139].

#### 5.3.3. Targeting Natural Antisense Transcripts

Natural antisense transcripts (NATs) are RNA molecules transcribed from the opposite DNA strand that share partial or full sequence complementarity with endogenous sense transcripts [29,140]. They serve as versatile regulators of gene expression across both eukaryotes and prokaryotes, with many genes producing long antisense transcripts that recruit chromatin repressors or interfere with transcription initiation [140]. Silencing or cleaving these NATs, potentially through Ago2, can relieve repression and could be the source of RNAa; however, as the Ago2 cleavage catalytic activity was reported to be dispensable for NAT targeting [33], the suggestion of a scaffolding role emerged as a more likely activation mechanism. NATs, however, are not universal features of all promoters, and saRNA-mediated activation has been reported in contexts where promoter-associated antisense transcripts have not been demonstrated [41]. Consequently, the presence of a NAT should not be considered a general prerequisite for saRNA design.

When a NAT-targeted approach is employed, the design strategy differs fundamentally from promoter-directed saRNA design [41]. Whereas promoter-targeted saRNAs are guided by genomic coordinates relative to the transcription start site, NAT-targeted design prioritizes the identification and accessibility of antisense RNA transcripts, making transcript-level annotation more critical than promoter position alone. A computational framework for this approach has been described in which the target gene’s genomic location, orientation, and transcriptional structure are first retrieved from annotation databases such as the UCSC RefSeq track [41]. Antisense transcripts are then identified from directional RNA databases, such as the UCSC Spliced EST track, using a hierarchy of four criteria applied as sequential filters: (a) transcripts that overlap the target’s promoter and the 5′ end of the target mRNA; (b) transcripts that overlap the target mRNA itself; (c) transcripts located 20–100 kb upstream of the target TSS; or (d) transcripts located 20–100 kb downstream of the target polyadenylation site. If antisense transcripts satisfying a higher-priority criterion are found, the remaining criteria are not evaluated. In the absence of identifiable spliced ESTs, the antisense genomic sequence flanking the TSS—typically 500 nucleotides upstream and downstream—can be used as an alternative target candidate, based on evidence that antisense RNAs are frequently found in regions surrounding transcription start sites.

Candidate saRNAs are then designed against the identified antisense target sequence using siRNA efficacy prediction algorithms. One such algorithm, GPboost, uses boosted genetic programming to predict oligonucleotide efficacy from sequence features alone and has demonstrated consistent performance across both antisense and siRNA datasets [11]. Following efficacy scoring, candidates are filtered to remove those containing homopolymer runs (aaaa, cccc, gggg, or uuuu) or having GC content outside the range of 20–55% [41]. This range differs from the 40–60% recommended for promoter-targeting saRNAs [10], reflecting the different study contexts from which these guidelines derive. An optional off-target filtering step removes candidates with a Hamming distance of less than two to any potential off-target transcript, and the remaining non-overlapping candidates are ranked by predicted knockdown efficacy, with the two highest-scoring saRNAs selected for a given antisense target.

## 6. Toward Rational saRNA Design

Review of the saRNA field reveals a large amount of data across different sources containing valuable information about the saRNA sequence, target location, promoter context, cell type, and activation efficiency. Rather than developing new design rules from a limited number of experiments, the data that already exists can be brought together to identify patterns that are not apparent when studies are considered individually.

### 6.1. Assembly of a Comprehensive and Annotated saRNA Database

Building on the work of Dar and Kumar [37], who published the first but no longer available database of saRNA in 2018, we compiled and curated a new, comprehensive list of reported and publicly available saRNA sequences. The database is available on Zenodo at the following link: https://doi.org/10.5281/zenodo.21871232.

Our database currently comprises 4706 entries from 114 source documents (93 journal articles, 20 patents and 1 preprint, published 2006–2026), describing 2424 unique saRNA sequences targeting 106 different genes. The difference accounts for several saRNA candidates being listed multiple times to include different testing conditions. Sequences were annotated where available or applicable with properties of the target (target gene, strand orientation and transcription start site [TSS]), genomic coordinates of the saRNA target site and its position relative to the TSS, the intrinsic sequence properties (length and GC content and modifications). We also list experimental conditions of each saRNA candidate tested, including cell type treated, concentrations used, transfection reagent used, and treatment duration. Importantly, the database provides author-reported saRNA activity assessment and assay used for activity readout. Studies typically report saRNA activity as the fold response of target gene mRNA expression in saRNA-treated cells compared to untreated control, as detected by quantitative PCR (qPCR). If not otherwise stated in the column “comments”, quantitative real-time PCR was used as the measurement method to acquire values in the column “effect reported”. Detailed descriptions of data collection and genomic annotations are provided in the Supplementary Information.

### 6.2. Opportunities for Artificial Intelligence-Assisted Design

Machine learning (ML) may provide an exciting opportunity to improve saRNA candidate selection by integrating multiple determinants of activity that are difficult to evaluate and account for using existing conventional design rules. The most realistic near-term application is likely to be the ranking of candidate saRNAs targeting a selected gene, enriching the experimental screens for active molecules and reducing the number of candidates that must be tested in vitro. Potential model inputs include saRNA sequence, its structural properties, distance from TSS, promoter architecture, transcription factor binding sites (TFBS), chromatin accessibility, basal gene expression, and cell-type-specific regulatory features. Such models could also help with the identification of feature combinations that are associated with activity and may even provide insight into mechanisms involved.

The challenges of applying ML to saRNA design are not unique to this field. Predicting siRNA efficacy from sequence has been an active area of ML research for more than a decade, supported by much larger datasets and more extensively characterized molecular mechanisms. Despite this, sequence-based siRNA efficacy prediction remains imperfect [141]. The saRNA field faces the same limitations: small datasets, heterogeneous assay conditions, and publication bias. All this is compounded by an incomplete understanding of the underlying mechanism and the added complexity of promoter and chromatin context. The siRNA experience suggests that progress will require not just larger datasets but also standardized activity measurements.

Sequence-based prediction alone is unlikely to be sufficient. Although existing design rules describe properties important for duplex stability, strand selection, and Ago2 loading, and may therefore help with exclusion of unsuitable molecules, they have limited ability to determine whether binding to a particular genomic site will induce transcription. Similarly, individual promoter annotations, such as distance from the TSS or the presence of (predicted) TFBS, may only be informative when interpreted within the broader regulatory and cell-specific context. Pretrained biological sequence models, such as DNABERT [142] and LucaOne [143], may help with the representation of complex sequence patterns and features compared to manually produced and selected features. Other recently developed models may also be relevant in this context. Evo2, which was trained on genomic sequences across a wide range of organisms and can process long sequence contexts, has been shown to capture regulatory and structural genomic features, such as TFBs [144]. More recently, models trained to predict gene regulatory features (TF occupancy, chromatin accessibility, gene expression levels) directly from DNA sequence have been developed [145]. These models encode aspects of promoter regulatory state that pure sequence models do not capture and may be more relevant in representing the genomic context on which saRNA activity depends. Their contribution is likely to lie in providing contextual feature representations rather than directly predicting saRNA activity.

The main limiting factor for ML-assisted saRNA design is currently the availability and structure of experimental data. Although our database contains several thousand saRNA records, these represent a comparatively small subset of independent genes and promoter regions, with many sequences being tested under different conditions. Another confounding variable is that the data are distributed across numerous cell lines, concentrations, transfection methods, and assay conditions. This makes it difficult to distinguish between sequence-dependent activity and gene-, cell-, and study-specific effects.

Publication bias represents a further obstacle. Each experimental screen generally contains many inactive candidates, whereas published studies preferentially report active molecules and frequently omit complete screening results. Consequently, literature-derived datasets are unbalanced, contain few negative examples, and do not accurately reflect the candidate distribution encountered during prospective design. Class balancing and model reweighting cannot compensate for the absence of these unreported data. The possibility that distinct molecular mechanisms underlie RNAa at different targets further complicates this picture: if the determinants of activity differ between mechanistic classes, a single model trained across all targets may fail to capture any of them adequately.

Reliable development and evaluation of future models will therefore require larger datasets containing complete panels, including inactive sequences, together with standardized activity measurements and experimental metadata. Predictive performance should be assessed on genes and promoters that were completely excluded from model training (leave-one-gene-out), since random separation of individual sequences (random 90/10 split) may overestimate generalizability. For practical candidate selection, enrichment of active saRNAs among highest-ranked candidates may also be more informative than overall classification accuracy.

At present, ML may therefore be best regarded as a tool for candidate prioritization, data integration and identification of potential design principles, rather than a standalone method for *de novo* saRNA prediction. Future predictive approaches will likely need to combine representations of saRNA sequence with genomic, epigenomic and cell-specific information. Continued expansion of experimentally validated datasets, improved annotation of cellular and promoter context, and a better understanding of RNAa mechanisms will be required before broadly transferable predictive models can be established. The updated saRNA database presented here may provide a foundation for such approaches and for guiding the systematic generation of the data required for future ML-assisted design.

## 7. Concluding Remarks

RNAa technology has created new opportunities for treating diseases caused by insufficient gene expression. saRNA has the potential to be used as a programmable drug to treat rare genetic diseases. However, in addition to common bottlenecks of delivery and stability, the prerequisite for that is also the establishment of robust and predictive design principles. Current publicly available design strategies can assist in prioritization of active candidates, but cannot reliably predict them, meaning that each new target requires costly empirical screening. This bottleneck is arguably the most significant constraint on the field’s broader translation.

The saRNA database, presented here, represents the most comprehensive, publicly available collection of reported saRNA sequences and associated experimental data to date. The annotating features in this database (activity, genomic location, and sequence properties) can already serve as descriptors for machine learning-assisted candidate prioritization and feature identification. A more meaningful prediction will, however, likely require a more standardized approach to activity reporting and complete screening panels (including the inactive sequences) that the current literature does not provide. Further investigation should clarify whether RNAa proceeds via a universal or heterogeneous molecular mechanism, in which case machine learning may assist in developing separate design principles and predictive models for each.

As a unified and continuously updated resource, our database will hopefully lay a foundation for developing robust and predictive saRNA design principles, support a machine learning-assisted prediction and selection of novel saRNA candidates, and ultimately help address the current lack of activatory RNA therapeutics. At the same time, saRNA is not alone in the expanding landscape of activatory RNA therapeutics. Complementary RNA-based approaches that upregulate gene expression at the post-transcriptional and translational levels are also clinically maturing. Antisense oligonucleotides targeting upstream open reading frames or translation inhibitory elements in 5′ untranslated regions can selectively increase protein production by relieving translational repression [146]. Several anti-miR oligonucleotides, which sequester endogenous miRNAs to de-repress their target transcripts, have reached clinical trials [147]. Anti-pre-miR approaches using peptide nucleic acids to block miRNA maturation offer another emerging route to rescuing expression of miRNA-suppressed genes [148]. While mechanistically distinct from RNAa, these strategies broaden the therapeutic toolkit for diseases caused by insufficient gene expression, and saRNA will need to establish its niche within this increasingly diverse landscape of activatory RNA modalities. Rational saRNA design, enabled by the unified saRNA sequence resource, could help maintain and build a competitive advantage for saRNA while perhaps also providing a foundation and inspiration for rational design strategies across the broader field of activatory RNA therapeutics.

## Data Availability

All data is provided within the paper and on Zenodo via the following DOI: https://doi.org/10.5281/zenodo.21871232. We also published a standalone website that provides a searchable presentation of the released data available via the following link: https://nic-sbi.github.io/sarna-database/. The accompanying project repository for the online database, including the annotation scripts documenting the Ensembl retrieval and exact-match coordinate mapping workflow, is available at https://github.com/NIC-SBI/sarna-database.

## Supplementary Materials

The following supporting information can be downloaded at: https://www.mdpi.com/article/10.3390/molecules31183249/s1, Section S1: Methods; Section S1.1: Data collection for the saRNA database; Section S1.2: Genomic annotations; Table S1: List of saRNA patents; Table S2: Overview of clinical trials evaluating saRNA-based therapeutics. References [47,48,52,58,59,60,61,62] are cited in the Supplementary Materials.

## Abbreviations

The following abbreviations are used in this manuscript:

RNAa	RNA activation
RNAi	RNA interference
saRNA	Small activating RNA
siRNA	Small interfering RNA
miRNA	Micro RNA
Ago	Argonaute
NAT	Natural antisense transcript
LncRNA	Long noncoding RNA
TSS	Transcription start site
RNAP II	RNA polymerase II
ASO	Antisense oligonucleotide
