# Supplementary material for: Small Activating RNAs: A Curated Database and an Overview of Rational Design Principles
Source: Molecules. 2026 Sep 14;31(18):3249. doi: 10.3390/molecules31183249 (PMC13609798; doi:10.3390/molecules31183249)
Supplement: Supplementary file 1 [file molecules-31-03249-s001.zip › molecules-4528862-supplementary.pdf]

Supplementary materials for manuscript

Small Activating RNAs: A Curated Database and an Overview of Rational Design Principles

Petra Sušjan-Leite, Peter Pečan, Ana Godeša, Fabienne Ramona Caldana and Roman Jerala

Methods

Data collection for the saRNA database

For data acquisition, curation and database development, we performed a literature search for saRNA-related keywords in research articles, patents and preprints. The following terms were used to construct PubMed queries: "saRNA," "RNA activation," "RNAa," "siRNA activation," "activating RNA," "antigene RNA," "agRNA" and "dsRNA-mediated transcriptional activation." Because the abbreviation "saRNA" is also used for self-amplifying RNA in the vaccine and replicon literature, results containing "self-amplifying RNA," "self-amplifying," or "replicon" were excluded during screening; approximately half of the initial "saRNA" hits referred to self-amplifying RNA rather than small activating RNA. A PubMed search combining the above terms and applying this exclusion returned approximately 540 records. Google Patents was searched using the same keyword set. The initial results were filtered manually to identify documents reporting original saRNA sequence data. We excluded reviews, non-English articles, conference abstracts, papers that did not report saRNA sequences or original experimental data and preprints published before 2025. Following these exclusions, 114 source documents — comprising 93 peer-reviewed journal articles, 20 patents and 1 preprint published between 2006 and 2026 — were retained for data extraction.

Each retained document was manually inspected, and saRNA-relevant fields were defined and extracted on a per-observation basis. Screening and data extraction were carried out by three curators; however, curators did not independently verify each other's work, and no formal inter-rater reliability assessment was performed. One row in the database represents one reported experimental observation; the same nucleotide sequence may therefore appear in multiple rows when it was tested under different conditions, in different models, or in different publications. In total, 4,706 data entries describing 2,424 unique saRNA sequences targeting 106 genes across 131 cell lines or model systems were compiled. The majority of records derive from human studies (4,530), with additional entries from murine (37), African green monkey (19), chimpanzee (16) and rat (12) models. Chemical modification information was reported for 3,627 of the 4,706 records.

Sequence identity (ID) is based on the normalized combination of sense and antisense sequence strings; chemical modifications, conjugates, delivery systems, concentration, publication, and other experimental conditions do not affect sequence ID. Overlapping publications were handled by retaining all source-reported observations as separate records linked through this shared sequence identifier. Of the 2,424 unique sequences, 135 appear in more than one publication and 113 appear in both a patent and a journal article. 70 of the 114source documents share at least one sequence with another document. This approach preserves the full provenance of each observation while enabling cross-referencing of sequences tested in multiple studies.

Activity values in the column ″effect_reported″ were preserved in their original reported format (fold changes, ranges, categorical descriptors or arbitrary units) and were not forced onto a common scoring scale, because the underlying assays and measurement definitions are heterogeneous across publications. Studies typically report saRNA activity as the fold-response of target gene mRNA expression in saRNA-treated cells compared to untreated control, as detected by quantitative real-time PCR. If not otherwise stated in the column ″comments″, this was the predominant measurement method used to acquire values in the column ″effect_reported″ . Other techniques reported include Western blot and fluorescence-based assays reporting arbitrary fluorescence intensity units. Column "comments" also includes other source-reported text which was added wherever practical.

Genomic annotations

saRNA genomic annotations provided in the saRNA database were generated using species-specific Ensembl gene models and reference assemblies. Gene metadata and sequence windows were retrieved through the Ensembl REST API using custom scripts deposited with the database workflow; the main Ensembl annotations correspond to Ensembl release 116 (June 2026), with human GRCh38 annotations based on GRCh38.p14/GCA_000001405.29. Human GRCh37 rescue mappings were generated through the separate Ensembl GRCh37 REST service, whose archive gene annotation is based on Ensembl release 75. For each genomic target, the Ensembl gene ID, chromosome, strand orientation, and transcription start site (TSS) were retrieved from Ensembl, using the canonical transcript TSS when available. saRNA sequences were converted to DNA sequence, terminal TT/UU overhangs were trimmed only when present on both strands, and exact matches were searched within a ±10 kb window around the TSS. The sense sequence, antisense sequence, and their reverse complements were searched. Coordinate-level annotation was assigned only for exact A/C/G/T nucleotide matches. Ambiguous or modified sequences were not force-mapped. If multiple exact matches were detected within the TSS window, the match with midpoint closest to the TSS was selected. Coordinates are reported as 1-based inclusive genomic intervals and as strand-aware positions relative to the TSS, with upstream positions shown as negative values. Human sequences were first aligned to the current standard reference assembly of the human genome – GRCh38 (Genome Reference Consortium Human Build 38). Rows that failed the exact alignment were additionally tested through a GRCh37/hg19 lookup and alignment workflow. Non-human sequences were aligned against the corresponding Ensembl species assemblies. The reference genome/assembly used for each coordinate assignment is explicitly provided in the database. Rows without exact sequence matches retain gene-level metadata where available but have blank coordinate-level fields. Genomic coordinates should be interpreted as computational exact-match annotations relative to species-specific Ensembl gene models, not as definitive experimental evidence of the active molecular target site. Canonical-transcript TSS selection may not capture the promoter or transcript isoform active in every experimental cell type, particularly for genes with alternative promoters or NAT-associated mechanisms.

Supplementary Tables

Table S1: List of saRNA patents.

Patent number	Title
US9297008B2	Small Activating Rna Molecules And Methods Of Use
EP3594348A1	C/ebp Alpha Short Activating Rna Compositions And Methods Of Use
WO2011112607A3	Nkx3-1 Sarna And Klf4 Sarna And Uses Thereof
US20130210146A1	Methods And Compositions For Generation Of Induced Pluripotent Stem Cells By RNAa
WO2011053994A1	Modulation of LDL Receptor gene expression
CN102657878A	Application of small activating RNA (Ribonucleic Acid) of INTS6 (Homo Sapiens Integrator Complex Subunit 6) gene to preparation of prostate cancer fighting medicament
US2015126578A1	Modified Small Activating RNA Molecules and Methods of Use
WO2016145608A1	SMALL ACTIVATING RNA, MANUFACTURING METHOD AND APPLICATION THEREOF / Small-activating RNA, preparation method and applications thereof
CN106244588A	SaRNA for activating expression of RUNX3 in lung cancer cell, and application of saRNA
AU2019252204A1	Novel small activating RNA
US2022064642A1	OLIGOMERIC NUCLEIC ACID MOLECULE AND APPLICATION THEREOF
US2022096350A1	OLIGONUCLEOTIDES FOR SKIN CARE
US2022096516A1	OLIGONUCLEOTIDE MOLECULE AND APPLICATION THEREOF IN TUMOR THERAPY
WO2022200810A1	TMEM173 SARNA COMPOSITIONS AND METHODS OF USE
WO2022215066A1	SMALL ACTIVATING RNA INCREASING SHANK EXPRESSION AND METHOD OF TREATING INTELLECTUAL DISABILITIES AND ASSOCIATED COMORBIDITIES ASSOCIATED WITH SHANK HAPLOINSUFFICIENCY
WO2023138502A1	OLIGONUCLEOTIDE MODULATORS ACTIVATING UTROPHIN EXPRESSION
WO2020221309A1	OLIGOMERIC NUCLEIC ACID MOLECULE, AND APPLICATION THEREOF IN ACUTE INTERMITTENT PORPHYRIA TREATMENT
US2023212565A1	NUCLEIC ACID MOLECULE FOR TREATING THROMBOCYTOPENIA AND APPLICATION THEREOF
WO2023170435A1	IL10 SARNA COMPOSITIONS AND METHODS OF USE

Table S2: Overview of clinical trials evaluating saRNA-based therapeutics.

Drug name	Name of the study	Target	Indication	Trial phase	Date of study start/ study completion	Registry identifier	Ref.
MTL-CEBPA	OUTREACH	*CEBPA*	Advanced hepatocellular carcinoma with cirrhosis	Phase 1a/b	1.03.2016/ 31.07.2025 (Estimated)	NCT02716012	*[47], [48]*
MTL-CEBPA + sorafenib	OUTREACH 2	*CEBPA*	Advanced liver cancer (hepatocellular carcinoma) as a result of hepatitis B and/or C infection	Phase 2	1.01.2022/ 25.01.2025	NCT04710641	.*[58]*
MTL-CEBPA + pembrolizumab	TIMEPOINT	*CEBPA*	Advanced solid tumors	Phase 1a/b	13.11.2019/ 25.01.2025	NCT04105335	*[59], [60]*
MTL-CEBPA + atezolizumab + bevacizumab	/	*CEBPA*	Advanced hepatocellular carcinoma (no prior systemic therapy)	Phase 1	2.08.2021/ Apr. 2027 (Estimated)	NCT05097911	*[61]*.
RAG-01	RAG-01	*p21*	Non-muscle-invasive Bladder Cancer (NMIBC) Who Have Failed Bacillus Calmette Guérin (BCG) Therapy	Phase 1	3.04.2024 /31.08.2028 (Estimated)	NCT06351904	*[62]*
RAG-18	RAG-18	*UTRN*	Duchenne Muscular Dystrophy (DMD)	Early Phase 1	12.12.2025/ Nov. 2026 (Estimated)	NCT07282652	*[52]*
